# A comparison of the image quality between deep learning reconstruction algorithm and iDose4 using low dose abdominopelvic computed tomography for individuals with normal BMI

**DOI:** 10.1177/20503121251336046

**Published:** 2025-08-22

**Authors:** Thejas Marike Shivakumar, Nitika C. Panakkal, Shailesh Nayak, Rajagopal Kadavigere, Tanushree R. Kamath, Suresh Sukumar

**Affiliations:** 1 Department of Medical Imaging Technology, Manipal College of Health Professions, Manipal Academy of Higher Education, Karnataka, India; 2 Radio-Diagnosis and Imaging, Department of Radio Diagnosis and Medical Imaging, Kasturba Medical College, Manipal Academy of Higher Education, Karnataka, India

**Keywords:** Image reconstruction, image quality, deep learning reconstruction, Precise Image reconstruction, computed tomography

## Abstract

**Objectives:**

Radiation exposure has been a cause of concern in computed tomography imaging. Reducing radiation dose increases the image noise which can be compensated by using reconstruction techniques. Recently artificial intelligence-based reconstruction technique has been introduced. Therefore, the purpose of the study was to prospectively compare the image quality between Idose4 and Precise Image in normal BMI individuals.

**Methods:**

Sixty-six consecutive patients with a normal body habitus undergoing contrast-enhanced abdomen and pelvis scan were included in the study. All scans were performed using 100 kVp and tube current modulation. The acquired images were reconstructed to iDose4 and precise imaging. Quantitatively images were analyzed by placing regions of interest in different organs to estimate the image noise, signal-to-noise ratio, and contrast-to-noise ratio. Qualitative analysis was done by two radiologists on a five-point Likert scale.

**Results:**

Image noise was significantly reduced using Precise Image across the plain (9.11 ± 1.43 vs 8.18 ± 1.2), arterial (14.34 ± 2.1 vs 10.21 ± 1.5), and portovenous phase (14.78 ± 2.30 vs 11.97 ± 2.07) with maximum noise reduction in the arterial and portovenous phases. Signal-to-noise ratio and contrast-to-noise ratio was significantly improved in all the organs across the plain, arterial, and portovenous phases. Qualitative analysis showed no significant difference between Idose4 and Precise Image with regards to visualization of large vessels in the arterial and portovenous phases. However, precise image was graded better than Idose4 with respect to visualization/conspicuity, image noise, and artifacts.

**Conclusion:**

Precise Image can be useful in reducing the image noise and improving the signal-to-noise ratio and contrast-to-noise ratio in low-dose computed tomography protocol among normal BMI individuals.

## Introduction

Since the 1970s, numerous developments in computed tomography (CT) hardware and software have been built resulting in the increased usage.^1^ Due to this increased demand, radiation exposure has become a cause of concern. However, implementing methods to reduce radiation dose often result in increased image noise and filtered back projection (FBP) reconstruction techniques are not suited for low-dose CTs.^2^ To maintain image quality at lower doses, iterative reconstruction (IR) technique was introduced. This technique increases the spatial resolution and decreases noise making it possible to reduce radiation dose by 23%–76%.^3,4^ The model-based IR (MBIR) that uses both forward and backward projection steps are computationally demanding requiring longer reconstruction time.^2^ Most of the CT vendors use a combined IR and FBP approach known as hybrid iterative reconstruction (HIR), that uses only a single backward projection phase to provide a compromise between the MBIR and FBP. All major vendors have their variations such as Adaptive statistical iterative reconstruction (ASiR), GE healthcare, Adaptive iterative dose reduction 3D (AIDR), Toshiba, Sinogram affirmed iterative reconstruction (SAFIRE) Siemens and iDose, Phillips.^5^ The iDose4 filters noise effectively in both projection and image domain and has reportedly brought about dose reduction of upto 76% without loss in image quality.^6^ Most recently, artificial intelligence (AI)-based deep learning reconstruction (DLR) techniques have been introduced to reduce noise levels and stimulate signals to deliver sharp, clear, and distinct images at faster speeds. This DLR makes use of convolutional neural networks (CNN) that is trained to reproduce noise patterns of conventional dose images from raw data of low-dose scans in a supervised learning process using simulation techniques.^7^ Different manufacturers have come up with their own AI reconstruction techniques namely AiCE by Cannon, TrueFidelity by GE, and Precise Image by Philips. A few studies have evaluated the usefulness of AI reconstruction techniques for improving image quality in terms of signal-to-noise ratio (SNR) and contrast-to-noise ratio (CNR) in different body regions.^3,8,9^ Much of the studies reported in literature have only estimated image noise and CNR in either the pre-contrast(plain), arterial phase or portovenous phase. Since deep learning technique involves training the algorithm using different datasets, evaluating the performance of AI algorithm in reducing noise in both pre- and postcontrast phases is essential. To our knowledge, no studies have been done to assess the image quality in abdomen and pelvis for the three phases including plain, arterial, and portovenous phases using precise image. Also, very few studies have evaluated the image quality of these AI-based reconstruction techniques at lower doses. Moreover, all the studies in literature have included a wide range of body habitus ranging from a lower to higher BMI. Body size can influence how radiation is distributed in body and impact image quality. Following a previous study that assessed the feasibility of using 100 kVp among normal BMI patients using iDose4, the current study sought to assess whether the DLR method could improve image quality as described in the literature.^10^ Therefore, the study aims to compare the image quality between Idose4 and AI-based DLR technique (precise image) using a lower kVp of 100 among patients with a normal body habitus for a multiphase abdomen and pelvis CT.

## Methods

The prospective cross-sectional study was conducted from September 2023 to January 2024 and received Institutional Ethics Committee approval (IEC no: 182/2023).

At 5% level of significance, assuming 80% power, the minimum sample size required to compare the means of image quality was calculated using the following formula $n\;=\;{\left(Z_{\alpha /2}\;+\;Z_{\beta }\right)}^{2}\;*\;2\;*\;\sigma^{\text{2}}/d^{\text{2}}$ , where n represents minimum number of subjects, σ^2^ represents population variance, and *d* represents the clinically significant difference.

After obtaining informed written consent, around 66 patients were referred for a contrast-enhanced computed tomography (CECT) of abdomen and pelvis and scanned using a 128-slice Incisive CT scanner. The technical parameter included were 100 kVp, pitch of 1.20, rotation time of 0.50, slice thickness of 5 mm, detector configuration 64*0.625, and tube current modulation with reference ranging from 80 to 250 mA. During the scan all patients were administered 80 ml of low osmolar contrast media Iohexol (Omnipaque 350) at a flow rate of 3.5 ml/s. The imaging protocol included acquisition of three phases: plain, arterial, and portovenous phases. All contrast scans were acquired with a delay of 8 and 45 s postthreshold for the arterial and portovenous phase, respectively. Images were reconstructed using iDose (level 4) and Precise Image (Standard). All the images were evaluated quantitatively and qualitatively.

### Patient selection

All patients with a normal BMI referred for a triple phase CECT abdomen and pelvis were included in the study. Patients with conditions like fatty liver, cirrhosis that could change the density of organs were excluded from the study.

### Quantitative analysis

**Figure 1.**
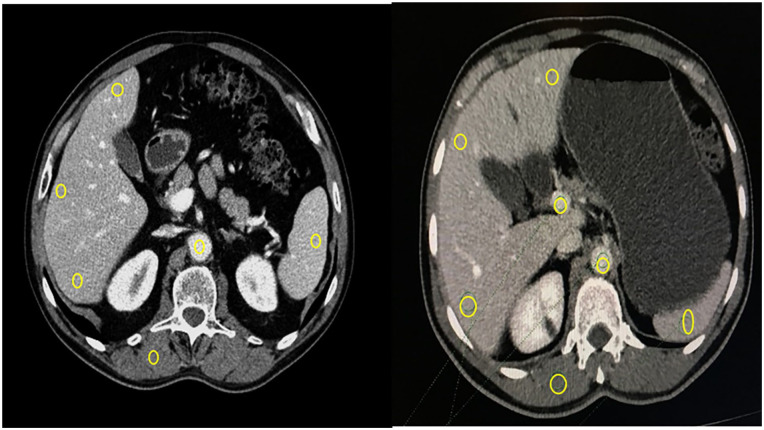
ROI placement in different organs. Figure adapted from “Optimising contrast volume based on body mass index in low tube voltage CT of abdomen and pelvis” by Panakkal N C. EPOS (online) ECR2025.10.26044/ecr2025/C-15098. https://epos.myesr.org/poster/esr/ecr2025/C-15098

For quantitative analysis, CT attenuation numbers were estimated by placing regions of interest (ROIs) in the liver, aorta, spleen, and posterior paraspinal muscle on the arterial, plain, and portovenous phases. Additionally, in portovenous phase, the ROI was drawn on the portal vein.^11^ The ROI area for vessels was 25 mm^2^ and for organs was 100 mm^2^ (Figure 1). ROIs were placed in the organs in two consecutive slices, and average CT Hounsfield numbers were taken to estimate image quality parameters such as SNR, CNR, and image noise using equations (1)–(3).^12,13^ Image noise was estimated by taking average of the standard deviation of all organs.

(1) $$CNR=\frac{CT\;number\;ROI-CT\;number\;ROI_{paraspinal\;Muscle}}{SD\;of\;ROI\;paraspinal\;muscle}$$

(2) $$SNR=\frac{CT\;number\;ROI_{Organ}}{SD_{Organ}}$$

(3) Imagenoise=SDoforgans

### Qualitative analysis

A blinded qualitative analysis was conducted by two board-certified radiologists. Image quality was graded on a 5-point Likert scale based on “Visualization/conspicuity (1-cannot identify; 2-suboptimal; 3-acceptable; 4-better than acceptable; 5-excellently visualized). Critical reproduction (1-blurry; 2-suboptimal; 3-acceptable; 4-better than acceptable; 5-sharpest). Visualization of large vessels (1-cannot identify; 2-suboptimal; 3-acceptable; 4-better than acceptable; 5- excellently visualized). Image contrast (1-very poor contrast; 2-suboptimal image contrast; 3-acceptable image contrast; 4-above-average contrast; 5-excellent image contrast). Image noise (1-un acceptable image noise; 2-above-average noise; 3-average image noise; 4-less-than-average noise; 5-minimal image noise).” Artifacts were assessed in a four-point Likert scale. “(1-Artifacts affecting diagnostic information, 2-Major artifacts affecting visualization of major structures but diagnosis still possible, 3-Minor artifacts are not interfering with the diagnostic decision, 4-No artifacts).”^14^

### Statistical analysis

The data were analyzed using International business machines corporation (IBM) Statistical package for the social sciences Statistics 21.0. Demographic characteristics and image quality parameters (CNR, SNR, and image noise) were summarized using descriptive statistics. To compare image quality between two techniques, paired *t*-test was used for normally distributed data and skewed data was analyzed using the Wilcoxon signed rank test. Intraclass correlation was done to evaluate rater agreement. A *p*-value of less than 0.05 was considered statistical significance.

## Results

### Patient characteristics

There were 66 patients included in the study with a normal body mass index between 18.9 and 24.9 kg/m^2^ with an average BMI of 22.8 ± 1.19, of which 35 were males and 31 were females. A wide range of age (18–88 years, mean 52 ± 17.5 years), height (150–185 cm, mean, 169.9 ± 8.2 cm), and weight (50–81 kg, mean 65.5 ± 8.0) were noted.

### Quantitative image quality

The mean CT numbers, SNR, CNR are presented in Tables 1 and 2. In the normal BMI group, CT numbers didn’t differ significantly between iDose4 and Precise Image across the three phases apart from the liver in plain images. Image noise was significantly reduced with precise image in the plain (9.1 ± 1.4 vs 8.1 ± 1.2; *p* < 0.001), arterial (14.3 ± 2.1 vs 10.2 ± 5; *p* < 0.001), and portovenous (14.7 ± 2.3 vs 11.9 ± 2; *p* < 0.001) phase. SNR was significantly improved in all the organs across the plain, arterial, and portovenous phases using precise image as shown in Table 1. However, the increase in SNR in the aorta in plain series was not significant. CNR was observed to significantly increase in all the organs across the plain, arterial, and portovenous phases using precise image. However, the increase in CNR in the liver in plain series was not significant as shown in Table 2.

**Table 1.** Descriptive summary of CT numbers in mean/median (Q1, Q3) for Idose4 and Precise Image across the plain, arterial, and portovenous phase.

Organs	Idose4	Precise image	*p*-Value
Plain			
Aorta	42.7 ± 4	42 (39.8, 45.5)	0.4
Liver	53 (47, 57.6)	53.2 (46.2, 56.7)	0.000
Spleen	49.7 (47.5, 52.1)	49.2 (47, 52.5)	0.5
Arterial			
Aorta	375.2 (333.6, 442.2)	381.2 (333, 442)	0.1
Liver	62.5 ± 11.6	62 ± 11.1	0.3
Spleen	115.1 ± 24.7	114.6 ± 24.7	0.3
Portovenous			
Aorta	175 ± 27.4	174.6 ± 27.3	0.5
Liver	114.9 ± 20.5	115.4 ± 20.3	0.5
Spleen	126 (113, 134.7)	126 (113, 138.1)	0.4
Portal vein	177 ± 27.2	178 ± 27.4	0.6

**Table 2.** Descriptive summary of CNR and SNR in mean/median (Q1, Q3) for Idose4 and Precise Image across the plain, arterial, and portovenous phase.

Phases	Organs	CNR		*p*-Value	SNR		*p*-Value
		Idose4	Precise image		Idose4	Precise image	
Plain	Aorta	0.71 (1.63, 1.26)	0.82 (0.26, 1.40)	<0.001	4.41 (5.07, 3.82)	4.51 (5.22, 4.00)	0.34
	Liver	0.35 (1.01, 0.33)	0.35 (0.99, 0.44)	0.367	6.30 ± 1.70	7.03 ± 1.65	<0.001
	Spleen	0.028 ± 0.923	0.0155 ± 0.94	<0.001	5.80 (6.88, 5.17)	6.75 (7.54, 5.98)	<0.001
Arterial phase	Aorta	25.72 (31.51, 22.80)	38.57 (47.81, 31.81)	<0.001	24.83 ± 8.09	36.24 ± 10.36	<0.001
	Liver	0.57 ± 1.10	0.74 ± 1.5	<0.001	5.11 ± 1.02	7.60 ± 1.51	<0.001
	Spleen	4.54 (6.15, 3.12)	7.05 (8.89, 4.03)	<0.001	4.62 (5.83, 3.66)	9.80 (12.2, 7.33)	<0.001
Porto venous	Aorta	8.47 (10.58, 6.73)	12.14 (14.42, 9.70)	<0.001	11.08 ± 3.38	15.86 ± 4.62	<0.001
	Liver	4.05 ± 1.80	5.85 ± 2.66	<0.001	6.12 (9.63, 5.25)	8.65 (9.66, 7.34)	0.039
	Spleen	4.94 (5.89, 3.89)	6.96 (8.50, 5.72)	<0.001	8.98 (11.35, 7.83)	14 (17.36, 11.97)	<0.001
	Portal vein	8.99 ± 2.68	12.90 ± 4.11	<0.001	11.14 ± 2.69	15.45 ± 3.61	<0.001

### Qualitative image quality

**Figure 2.**
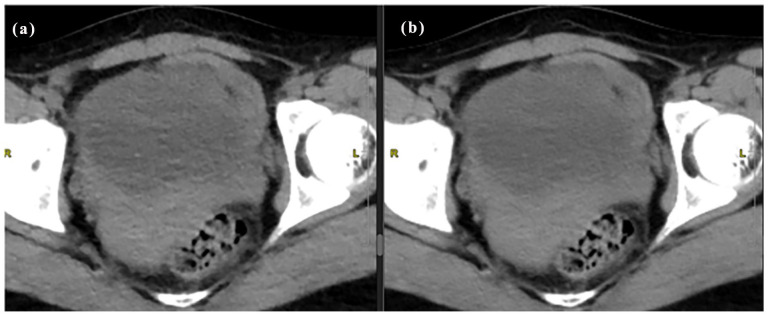
Idose4 (a) versus Precise Image (b) for unenhanced abdomen and pelvis CT.

**Figure 3.**
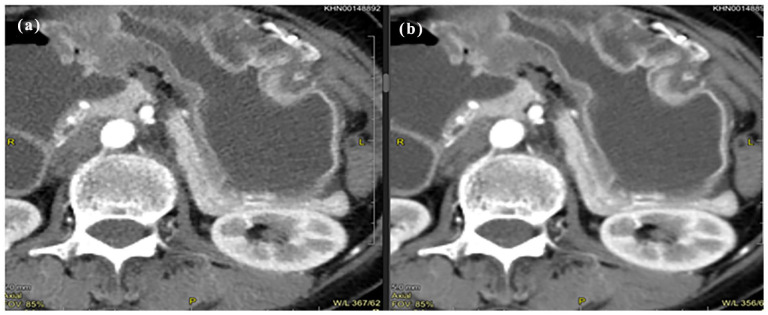
Idose4 (a) versus Precise Image (b) for arterial phase abdomen and pelvis CT.

**Figure 4.**
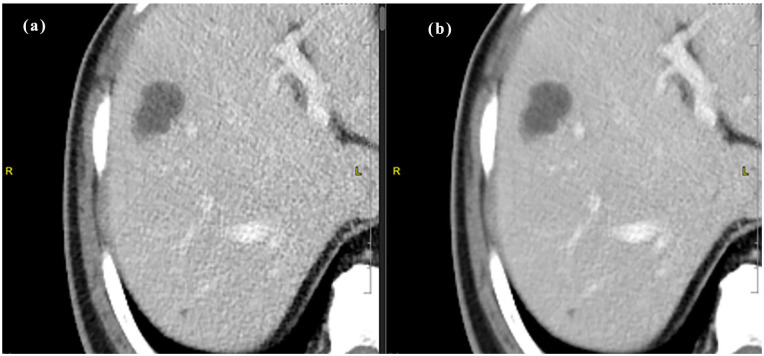
Idose4 (a) versus Precise Image (b) for portal venous phase abdomen and pelvis CT.

The intraclass correlation agreement between the readers ranged from 0.916 to 0.988 indicating excellent reliability. Most of the images in iDose4 were graded excellent across the plain, arterial, and portovenous phases with respect to critical reproduction (89%, 90%, and 81%), visualization of large vessels (86%), and image contrast (93%, 96%, and 90%) as shown in Table 3. Also, there was no significant difference in image quality between Idose4 and Precise Image with respect to visualization of large vessels in the arterial and portovenous phases. Around 20% of the Idose4 images were graded 3 with respect to critical reproduction and approximately 0.06% graded 3 with regards to image contrast for the plain phase indicating acceptable sharpness and image contrast. Nevertheless, there was a significant improvement in image quality in these categories using Precise Image (Table 4). Precise Image was also graded better than iDose4 with respect to visualization/conspicuity, image noise, and artifacts as shown in Figures 2, 3, and 4. Most of the images in precise were graded 4 and 5 in all image quality criteria indicating better/excellent image quality.

**Table 3.** Qualitative assessment of image quality scores for both raters in Idose4 and Precise Image.

Image quality criteria	Idose4			Precise image		
	Plain	Arterial	PV	Plain	Arterial	PV
Visualization/conspicuity	0/0/6/53/7	0/0/4/59/3	0/0/0/59/7	0/0/0/7/59	0/0/0/6/60	0/0/0/4/62
	0/0/6/52/8	0/0/4/58/4	0/0/0/58/6	0/0/0/6/58	0/0/0/7/59	0/0/0/3/63
Critical reproduction	0/0/7/0/59	0/0/0/6/60	0/0/0/12/54	0/0/0/7/59	0/0/0/0/66	0/0/0/3/63
	0/0/7/1/58	0/0/0/5/61	0/0/0/13/53	0/0/0/6/58	0/0/0/1/65	0/0/0/4/62
Visualization of large vessels	0/0/0/9/57	0/0/0/9/57	0/0/0/9/57	0/0/0/8/58	0/0/0/2/64	0/0/0/2/64
	0/0/0/8/58	0/0/0/8/58	0/0/0/8/58	0/0/0/7/59	0/0/0/1/65	0/0/0/3/63
Image contrast	0/0/4/0/62	0/0/2/0/64	0/0/2/4/60	0/0/1/3/62	0/0/0/2/64	0/0/0/6/60
	0/0/3/1/62	0/0/1/0/65	0/0/2/5/59	0/0/0/4/62	0/0/0/3/63	0/0/0/5/61
Image noise	0/0/9/57/0	0/0/9/57/0	0/0/9/57/0	0/0/1/8/57	0/0/0/9/57	0/0/0/13/53
	0/0/11/55/0	0/0/10/58/0	0/0/8/58/0	0/0/0/9/57	0/0/0/10/58	0/0/0/12/54
Artifacts	0/4/56/6	0/4/56/6	0/9/57/0	0/0/10/56	0/0/9/57	0/0/13/52
	0/3/57/6	0/3/57/6	0/8/56/0	0/0/9/57	0/0/8/58	0/0/12/53

**Table 4.** Comparison of qualitative image quality parameters between Idose4 and Precise Image.

Qualitative parameters	Plain			Arterial			Portovenous		
	Idose 4	Precise	*p*-Value	Idose 4	Precise	*p*-Value	Idose 4	Precise	*p*-Value
Visualization/conspicuity	4 ± 0.4	4.8 ± 0.3	<0.001	4 ± 0.2	4.9 ± 0.1	<0.001	4.2 ± 0.3	4.9 ± 0.1	<0.001
Critical reproduction	4.7 ± 0.6	4.9 ± 0.3	<0.001	4.7 ± 0.6	4.9 ± 0.1	<0.001	4.7 ± 0.1	4.9 ± 0.1	<0.001
Visualization of large vessels	4.7 ± 0.3	4.9 ± 0.2	<0.001	4.8 ± 0.3	4.8 ± 0.08	0.973	4.8 ± 0.3	4.9 ± 0.3	0.08
Image contrast	4.7 ± 0.4	4.9 ± 0.2	<0.001	4.7 ± 0.1	4.9 ± 0.08	<0.001	4.7 ± 0.2	4.9 ± 0.1	<0.001
Image noise	3.8 ± 0.3	4.8 ± 0.3	<0.001	3.8 ± 0.3	4.8 ± 0.3	<0.001	3.8 ± 0.3	4.8 ± 0.3	<0.001
Artifacts	3 ± 0.3	3.4 ± 0.1	<0.001	2.8 ± 0.3	3.4 ± 0.1	<0.001	2.8 ± 0.3	3.4 ± 0.2	<0.001

## Discussion

A lot of studies in the literature shows improvement in image quality using AI-based reconstruction techniques.^8,9,15–20^ Some of these studies have been done on phantom and results may have to be validated with clinical studies.^15,16^ Different manufacturers have introduced their own deep learning algorithms that make use of deep CNNs into the image reconstruction process. A majority of studies reported, have evaluated image quality using the TrueFidelity by GE healthcare and AiCE by canon medical systems. The studies reported an improvement in SNR and CNR compared to IR, hybrid reconstruction MBIR techniques.^19^ Recently, Philips healthcare introduced their DLR named Precise Image that has been tested on both phantoms and patients to provide improved image quality. Phantom study conducted by Greffier et al.^15^ reported a reduction in noise using standard and smoother levels. Similar studies conducted by Greffier et al.^7^ reported a significant improvement in the overall image quality subjectively for liver metastasis conspicuity using smooth and smoother levels. The results of the present study also reported a better image quality both subjectively and objectively in terms of SNR, CNR, and reduced noise using DLR technique for multiphase CT abdomen and pelvis studies. However, a unique aspect of this study was that a low kVp was implemented in all three phases of the CECT abdomen and pelvis as opposed to 120 kVp used in all other studies reported in literature.^8,9,15–19^ In this regard, an image quality improvement is possible at low doses when a lower kVp is used in conjunction with precise imaging. Also, in all the studies reported, a wide range of body sizes were included, since body size is an important parameter influencing the absorption and distribution of radiation in the body and can therefore affect the image quality, the present study included only patient with a normal body mass index. Although, Tamura et al.^21^ found no differences in noise across the BMI groups, the results cannot be generalized to other vendors since the DLR algorithm used in their study was AiCE, and the present study used precise imaging.

In the present study, image noise was found to be significantly reduced using Precise Image. Similarly, SNR and CNR was significantly improved for all organs in all the phases. It was also observed that the maximum noise reduction and improvement of SNR and CNR occurred in the postcontrast phases (arterial and portovenous) compared to the precontrast phase. This significant improvement in image quality in contrast-enhanced images may be attributed to better differentiation of anatomical structures that allows DLR models to effectively identify and reduce noise. Tables 5 and 6 summarizes the image noise, and SNR and CNR values obtained in the present study and other studies reported in the literature that have used DLR technique for CT abdomen and pelvis. The results of the present study were comparable with the other studies reported. These variations can be explained due to the difference in exposure parameter used, protocol variations like scan delay, slice thickness.

**Table 5.** Comparison of image noise values in present and other studies reported in literature.

Technical parameters/scan series	Present study	Singh et al.^18^	Akagi et al.^19^	Ichikawa et al.^9^	Jensen et al.^17^
DLR algorithm	Precise image	AiCE	AiCE	TrueFidelity	TrueFidelity (DLIR-Low)
kVp	100	120	120	120	120
Plain	9.1	—	—	5.2	—
Arterial	14.3	—	13.9		—
Porto venous	14.7	11	14.6		9.9

**Table 6.** Comparison of CNR and SNR values in present and other studies reported in literature.

	Organs	Present study		Ichikawa et al.^9^		Jensen et al.^17^		Singh et al.^18^		Akagi et al.^19^	
Scan Series		CNR	SNR	CNR	SNR	CNR	SNR	CNR	SNR	CNR	SNR
Plain	Aorta	0.82	4.5	—	—	—	—	—	—	—	—
	Liver	0.35	7.03	—	9.6	—	—	—	—	—	—
Arterial	Aorta	38.57	36.2	—	—	—	—	—	—	19.9	—
	Liver	0.74	7.6	1	—	—	—	—	—	1.2	—
Porto venous	Aorta	12.1	15.8	—	15	14.3	—	—	—	3.3	—
	Liver	5.8	8.6	—	—	6.54	—	—	9.9	2	—
	Spleen	6.9	14	—	—	9.3	—	—	—	—	—
	Portal vein	12.9	15.4	—	—	—	—	—	—	3.4	—

Also, there was also a difference on how image noise was calculated in different studies. The present study estimated the image noise from the mean of standard deviation of all organs (aorta, liver, portal vein, and spleen), whereas study conducted by Akagi et al.^19^ estimated noise from standard deviation measured in paraspinal muscle. Similarly, Jensen et al.^17^ estimated the image noise from standard deviation of subcutaneous fat. A few studies estimated image noise from the standard deviation in liver parenchyma.^9,18^ Other factors like type of DLR used and strength of DLR can also influence noise reduction ability and noise texture.

This study has some limitations. First, only the standard filter of Precise Image DLR technique was considered, and other filters such as “smooth” and “smoother” were not compared. Second, although qualitative analysis showed promising results, prospective studies including different pathology as per clinical indication can be done to improve the clinical benefit. Also, evaluation for lesion conspicuity between both reconstruction algorithms were not performed due to limited cases. Therefore, future studies can be done to evaluate the performance of precise image in lesion conspicuity. The present study included only normal BMI individuals, since body size can be important parameter to determine image quality, further studies on different BMI ranges can be explored. The present study could only objectively evaluate the image quality indexes like SNR, CNR, and image noise. A more detailed method would include evaluating noise textures that correlates between adjacent pixels that influence the detectability of certain pathologies ideal for nonlinear algorithms like IR and DLR.^22,23^ Noise texture in the images can be calculated using the noise power spectrum by plotting the amplitudes with the frequency of image using Fourier transform.^24^ Considering this, further studies can be conducted to assess the effectiveness of DLR technique in different body sized individuals. Nevertheless, the use of widely popular image matrices such as SNR, CNR, and Image noise are still used frequently to evaluate the image quality, compare performance of modalities and quantitative evaluation of lesion detectability as it can be measured easily.^21,25,26^

## Conclusion

Compared to Idose4, the Precise Image reconstruction algorithm significantly decreased image noise and improved SNR and CNR. Qualitative analysis also reported favorable to the Precise Image reconstruction. Therefore, precise imaging may be useful in improving the image quality in low-dose abdomen and pelvis CT among normal BMI individuals.
